# Leptomeningeal Metastases: New Opportunities in the Modern Era

**DOI:** 10.1007/s13311-022-01261-4

**Published:** 2022-07-05

**Authors:** Jessica A. Wilcox, Min Jun Li, Adrienne A. Boire

**Affiliations:** 1 Department of Neurology, Memorial Sloan Kettering Cancer Center, New York, NY USA; 2 Brain Tumor Center, Human Oncology and Pathogenesis Program, Memorial Sloan Kettering Cancer Center, New York, NY USA

**Keywords:** Leptomeningeal metastases, Leptomeningeal disease, Central nervous system metastases, Brain metastases, Cerebrospinal fluid, Intrathecal therapy, Craniospinal radiation

## Abstract

Leptomeningeal metastases arise from cancer cell entry into the subarachnoid space, inflicting significant neurologic morbidity and mortality across a wide range of malignancies. The modern era of cancer therapeutics has seen an explosion of molecular-targeting agents and immune-mediated strategies for patients with breast, lung, and melanoma malignancies, with meaningful extracranial disease control and improvement in patient survival. However, the clinical efficacy of these agents in those with leptomeningeal metastases remains understudied, due to the relative rarity of this patient population, the investigational challenges associated with studying this dynamic disease state, and brisk disease pace. Nevertheless, retrospective studies, post hoc analyses, and small prospective trials in the last two decades provide a glimmer of hope for patients with leptomeningeal metastases, suggesting that several cancer-directed strategies are not only active in the intrathecal space but also improve survival against historical odds. The continued development of clinical trials devoted to patients with leptomeningeal metastases is critical to establish robust efficacy outcomes in this patient population, define drug pharmacokinetics in the intrathecal space, and uncover new avenues for treatment in the face of leptomeningeal therapeutic resistance.

**Supplementary Information:**

The online version contains supplementary material available at 10.1007/s13311-022-01261-4.

## Introduction

Leptomeningeal metastases (LM) represent an aggressive, advanced stage of cancer with substantial neurologic morbidity and a grim survival of 2–5 months [1, 2]. Also known as leptomeningeal carcinomatosis or neoplastic meningitis, LM occurs when cancer cells gain access to the cerebrospinal fluid (CSF) that surrounds the brain and spinal cord, either through hematogenous dissemination via the choroid plexus, retrograde venous extension, or direct seeding from parenchymal metastases across the glia limitans [3–5]. Upon entry, LM adopts two phenotypic states: adherent plaques to the brain and spinal cord visualized with contrast-enhanced magnetic resonance imaging (MRI), and free-floating cells within the spinal fluid detected via CSF cytology [6]. As such, diagnosis and surveillance of LM require full neuraxial MRI and CSF analysis to fully stage the burden of disease [7, 8]. The estimated incidence of LM from systemic malignancies is approximately 5–20% based on population and autopsy data [9–12]; the true incidence of LM is likely higher owing to insensitivity of CSF cytology and asymptomatic leptomeningeal seeding [13].

**Fig. 1:**
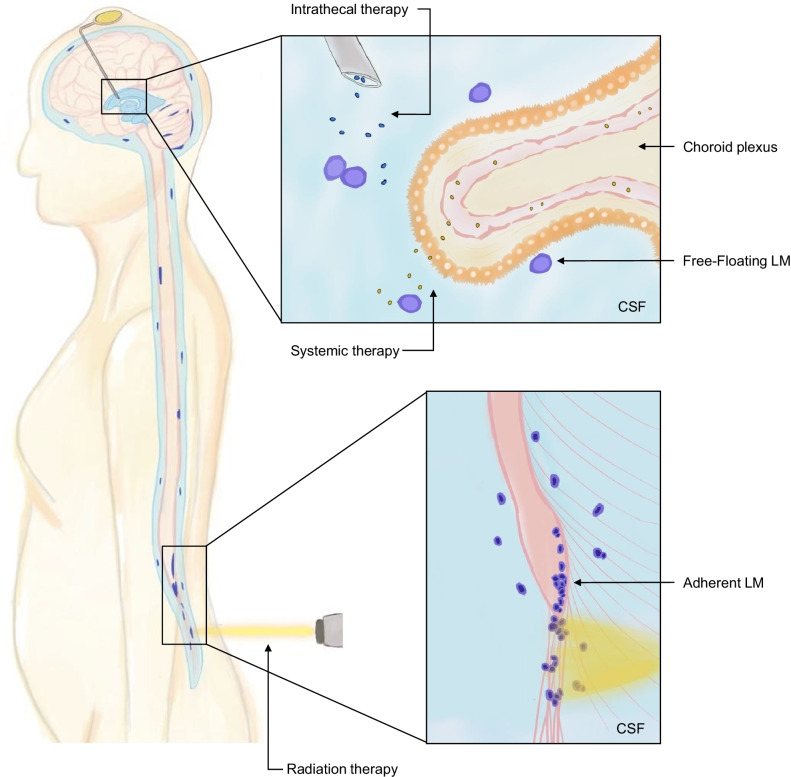
Anatomic considerations and conventional therapeutic strategies for leptomeningeal metastases. This illustration outlines the two phenotypic states of leptomeningeal metastases (LM), existing as both free-floating disseminated cells and adherent plaques to the brain and spinal cord. The three pillars of conventional therapies are shown, including intrathecal therapy (blue dots) through an Ommaya reservoir, systemic therapy (yellow dots) entering the cerebrospinal fluid (CSF) via the blood-CSF barrier of the choroid plexus, and involved-field radiation therapy (yellow beam) directed here against the adherent cells of the conus medullaris and cauda equina

Conventional treatment of LM includes intrathecal chemotherapy for those with adequate performance status, whole brain radiation therapy (WBRT) to cranial disease and/or involved-field radiation therapy (RT) to disease of the spinal cord and cauda equina, and systemic chemotherapies with adequate central nervous system (CNS) penetration (Fig. 1). Symptoms referrable to increased intracranial pressure can be ameliorated with palliative ventriculoperitoneal shunting [14]. For those without abnormal CSF flow dynamics, commonly used intrathecal therapy options include methotrexate [15–17], thiotepa [18], and topotecan [19]. No single intrathecal chemotherapy has emerged as providing a significant survival benefit, and their use is associated with increased neurotoxicity risk [20–22].

While modern targeted therapies and immunotherapy have resulted in meaningful prolongation of survival among certain cancer subtypes, the influence of such agents in the treatment of LM remains largely unexplored. Several challenges explain this investigational discordance. Patients with CNS metastases have long been excluded from clinical trials, perpetuating a pattern of poor outcomes and limited evidence-based data for this patient population. Part of this discrepancy lies in uncertainty regarding drug access to different CNS compartments. The blood–brain barrier (BBB) and blood-CSF barrier (BCSFB) represent two distinct vascular niches with differential pharmacologic permeability [23]; dedicated CSF pharmacokinetic monitoring is essential to understand access and durability of novel drugs into the intrathecal space. Recent neuro-oncologic advocacy has resulted in additional investigational protocols for patients with parenchymal brain metastases; however, LM overwhelmingly remains an exclusion criterion. Furthermore, genetic divergence between brain and extracranial sites of cancer highlights an important mechanism of therapeutic resistance, and intracranial sampling can uncover targetable, oncogenic drivers that are distinct from primary tumor [24, 25]. Basket trials matching intracranial oncogenes with targeted agents aim to capitalize on this principle for those with parenchymal brain metastases (NCT03994796). Genomic characterization of CSF in patients with LM may be similarly capable of providing therapeutic insights [26–28]; however, the clinical relevance of this data has not yet been recapitulated in prospective trials. As a result of these limitations, evidence supporting the use of modern targeted therapeutics in patients with LM relies heavily on retrospective reports, post hoc analyses, and small prospective studies (Table 1). Nevertheless, these insights collectively shed light on opportunity for this patient population and have inspired several novel LM-directed therapies currently in development (Table 2).

**Table 1:** Prospective clinical trials including patients with solid tumor LM

**Name**	**Type**	**Treatment**	***N***^**a**^	**Population**	**Median OS**^b^	**Other results**
Hitchins et al. [17]	RCT	IT MTX versus IT MTX + araC	44	SCLC LM = 13; breast LM = 11; adenocarcinoma unknown primary LM = 6; mixed histologies NOS = 13	IT MTX: 2.8 months IT MTX + araC: 1.6 months	IT MTX response rate = 61% IT MTX + araC response rate = 45% Response defined as CR, PR, SD
Grossman et al. [15]	RCT	IT MTX versus IT Thiotepa	52	Breast LM = 25; lung LM = 12; lymphoma LM = 10; mixed histologies NOS = 5	IT MTX: 3.7 months IT Thiotepa: 3.2 months	IT MTX SD = 32.1% IT Thiotepa SD = 12.5% No patients with CR or improvement Neurologic deterioration in 75% within 8 weeks of therapy
Glantz et al. [16]	RCT	IT DepoCyt versus IT MTX	61	Breast LM = 22; NSCLC LM = 6; melanoma LM = 5; SCLC LM = 4; mixed histologies NOS = 24	IT DepoCyt: 3.5 months IT MTX: 2.6 months	IT DepoCyt response rate = 26%, median TTP = 58 days IT MTX response rate = 20%, median TTP = 30 days Response defined as conversion to negative cytology plus stable or improved examination
Boogerd et al. [21]	RCT	PBC + / − ITC (MTX, araC)	35	Breast LM	ITC: 4.2 months No ITC: 7.0 months	ITC median TTP = 23 weeks No ITC median TTP = 24 weeks Neurologic toxicity in 47% ITC and 6% no-ITC patients
Groves et al. [19]	Phase II	IT Topotecan	62	Breast LM = 19; lung LM = 13; primary CNS LM = 14; mixed histologies NOS = 16	3.5 months	Median TTP = 7 weeks Improved or stable LM in 46.8%
Jackman et al. [62]	Phase I	High-dose gefitinib (750–1000 mg/day) × 2 weeks + maintenance gefitinib (500 mg/day) × 2 weeks	7	EGFR-mutant NSCLC LM	3.5 months	Median neurologic PFS = 2.3 months
Tamiya et al. [63]	Prospective	Afatinib 40 mg daily	11	EGFR-mutant NSCLC LM	3.8 months	ORR = 27.3% Median PFS = 2.0 months
Long et al. [166]	Phase II	Nivolumab 3 mg/kg q2 weeks	4	Melanoma LM	N/A	0 patients with LM had an intracranial response
Nanjo et al. [68]	Prospective	Osimertinib 80 mg daily	13	EGFR T790M-mutant NSCLC LM	NR	Median PFS = 7.2 months
Freedman et al. [103]	Phase II	Neratinib 240 mg daily + capecitabine 750 mg/m2 BID (14 days on/7 days off)	3	HER2+ breast LM	N/A	1 PR for 7 cycles; 1 SD for 4 cycles
Morikawa et al. [104]	Phase I	High-dose lapatinib (1000–2000 mg BID) days 1–3 and 15–17 + capecitabine 1500 mg BID days 8–14 and 22–28	4	HER2+ breast LM	N/A	1 PR; 1 SD
Yang et al. [66]	Phase I	Osimertinib 160 mg daily	41	EGFR-mutant NSCLC LM	11.0 months	LM ORR = 62% Median PFS = 8.6 months
Park et al. [67]	Phase II	Osimertinib 160 mg daily	40	EGFR T790M-mutant NSCLC LM	13.3 months	Intracranial DCR = 92.5% Median PFS = 8.0 months
Bauer et al. [83]	Phase II	Lorlatinib 100 mg daily	2	ALK+ NSCLC LM	N/A	1 intracranial CR with TTP of 21.9 months; 1 intracranial PR with TTP of 11.0 months
Le Rhun et al. [20]	Phase III	PBC + / − IT liposomal araC	73	Breast LM	IT araC: 7.3 months No IT araC: 4.0 months	IT araC median LM-PFS = 3.8 months No IT araC median LM-PFS = 2.2 months
Tolaney et al. [132]	Phase II	Abemaciclib 150–200 mg BID	10	HR+ HER2- breast LM = 7; HR+ HER2+ breast LM = 3	HER2-: 8.4 months	Intracranial ORR = 0% for HER2- and HER2+ LM Intracranial DCR = 28.6% for HER2- LM Intracranial DCR = 33.3% for HER2+ LM Median PFS = 5.9 months for HER2- LM
Kumthekar et al. [149]	Phase II	ANG1005 (paclitaxel trevatide) 600 mg/m2 q3 weeks	28	Breast LM	8.0 months	Intracranial ORR = 17% Intracranial CBR = 79% Median intracranial PFS = 14.9 weeks
Brastianos et al. [50]	Phase II	Pembrolizumab 200 mg q3 weeks	20	Breast LM = 17; lung LM = 2; ovarian LM = 1	3.6 months	Median intracranial PFS = 2.6 months 60% alive at 3 months
Brastianos et al. [51]	Phase II	Ipilimumab + Nivolumab q2-6 weeks^c^	18	Breast LM = 8; melanoma LM = 2; mixed histologies NOS = 8	2.9 months	Median intracranial PFS = 1.9 months 44% alive at 3 months
Yang et al. [36]	Phase I	Proton CSI 30 Gy in 10 fractions	21	NSCLC LM = 11; breast LM = 7; mixed histologies NOS = 3	8 months	Median CNS PFS = 7 months CNS response to therapy 100% at 1 month, 86% at 3 months, 63% at 6 months

*LM* leptomeningeal metastases, *OS* overall survival, *RCT* randomized controlled trial, *IT* intrathecal, *MTX* methotrexate, *araC* cytarabine, *SCLC* small cell lung cancer, *NOS* not otherwise specified, *CR* complete response, *PR* partial response, *SD* stable disease, *DepoCyt* sustained-release cytarabine, *NSCLC* non-small cell lung cancer, *TTP* time to progression, *PBC* physician’s best choice therapy, *ITC* intrathecal chemotherapy, *CNS* central nervous system, *EGFR* epidermal growth factor receptor, *PFS* progression free survival, *ORR* objective response rate, *N/A* not applicable, *NR* not reached, *HER2* human epidermal growth factor receptor 2, *DCR* disease control rate, *ALK* anaplastic lymphoma kinase, *CBR* clinical benefit rate, *CSI* craniospinal irradiation

^a^Number of total evaluable patients

^b^Survival data converted to months for uniformity

^c^Dosing and frequency for each specific tumor histology as per manufacturer’s guidelines

**Table 2:** Active therapeutic clinical trials for adults with solid tumor LM

NCT number^a^	Phase	*N*	Population	Treatment
NCT04543188	I	225	BRAF V600-mutant solid tumor	PF-07284890 (ARRY-461) + binimetinib
NCT03696030	I	39	HER2+ solid tumor	IT HER2 CAR T cells
NCT03025256	I	50	Melanoma	IV + IT nivolumab
NCT03719768	I	23	Solid tumor	Avelumab + WBRT
NCT05112549	I	46	Solid tumor	IT nivolumab
NCT04192981	I	36	PIK3CA-mutant solid tumor	GDC-0084 (paxalisib) + WBRT
NCT05184816	I	35	Solid tumor	IT deferoxamine
NCT03661424	I	16	HER2+ breast	Bi-specific antibody armed activated T cells (HER2 BATs)
NCT05034497	I	18	Solid tumor	IT rhenium-186 NanoLiposome
NCT04509596	I	94	HER2+ breast	DZD1516 + trastuzumab/capecitabine or trastuzumab emtansine
NCT03711422	I	25	EGFR-mutant NSCLC	Afatinib
NCT04197934	I	72	EGFR-mutant solid tumor	WSD0922-FU
NCT04588545	I/II	39	HER2+ breast	IT trastuzumab/pertuzumab + RT
NCT04511013	II	112	BRAF V600-mutant melanoma	Encorafenib/binimetinib/nivolumab versus ipilimumab/nivolumab
NCT04965090	II	40	EGFR-mutant NSCLC	Amivantamab + lazertinib
NCT03501979	II	30	HER2+ breast	Tucatinib + trastuzumab + capecitabine
NCT04420598	II	39	HER2+ or HER2-low breast	Trastuzumab deruxtecan
NCT04343573	II	111	Solid tumor	Proton CSI versus involved field RT
NCT04729348	II	19	Solid tumor	Pembrolizumab + lenvatinib
NCT04425681	II	20	EGFR-mutant NSCLC	Osimertinib + bevacizumab
NCT04233021	II	113	EGFR-mutant NSCLC	Osimertinib
NCT02422641	II	16	Breast	High-dose MTX
NCT04833205	II	30	EGFR-mutant NSCLC	Nimotuzumab + EGFR-TKI
NCT05146219	II	60	EGFR-mutant NSCLC	TY-9591
NCT02886585	II	102	Solid tumor	Pembrolizumab
NCT03613181	III	150	HER2- breast	ANG1005 (paclitaxel trevatide) versus PBC
NCT04356118	IV	30	NSCLC	Recombinant human endostatin + IT MTX + targeted agents
NCT04356222	IV	30	NSCLC	Durvalumab + IT MTX
NCT04944069	N/A	69	EGFR-mutant NSCLC	Almonertinib + bevacizumab
NCT04778800	N/A	60	EGFR-mutant NSCLC	Almonertinib

*LM* leptomeningeal metastases, *HER2* human epidermal growth factor receptor 2, *IT* intrathecal, *CAR* chimeric antigen receptor, *IV* intravenous, *WBRT* whole brain radiation therapy, *EGFR* epidermal growth factor receptor, *NSCLC* non-small cell lung cancer, *RT* radiation therapy, *CSI* craniospinal irradiation, *MTX* methotrexate, *TKI* tyrosine kinase inhibitor, *PBC* physician’s best choice, *N/A* not applicable or not listed

^a^Active, therapeutic, recruiting and not recruiting protocols registered on ClinicalTrials.gov as of February 28, 2022. Primary CNS malignancy-only and non-therapeutic protocols excluded

## Radiation Therapy

RT has long been one of the pillars of treatment of LM, typically in the form of WBRT for cranial and involved-field RT for spinal sites of disease [29]. Such strategies aim to provide both palliative and therapeutic effect, primarily to regions of bulky, adherent LM that are less amenable to systemic or intrathecal therapies [30, 31]. However, persistence of dynamic, suspended cancer cells throughout the intrathecal compartment limits the therapeutic durability of focal RT. Without simultaneous inclusion of CSF-penetrant systemic therapies to target disseminated disease, focally radiated sites are at a great risk of reseeding, and the “cytoreduction” benefit afforded by this approach is minimal. Consequently, survival benefit of conventional RT for LM has not been demonstrated in prospective studies, and observational studies are mixed with respect to the prognostic role of WBRT [31].

Unlike involved-field RT with its inherent risks of leaving sites of the CNS untreated, craniospinal irradiation (CSI) offers the ability to radiate the entire neuraxial compartment. The efficacy of photon CSI has been established in many pediatric tumors with leptomeningeal dissemination, such as medulloblastoma, ependymoma, and select hematologic malignancies [31]. However, photon CSI is not without considerable short-term and long-term risks related to exposure of the entire spinal column and visceral organs to radiation, namely myelosuppression, mucositis, endocrine dysfunction, stunted growth in children, and neurotoxicity [32]. These side effects limit the widespread applicability of photon CSI to adults with solid tumor LM, despite potential palliative benefits [33].

Proton craniospinal irradiation (pCSI) largely circumvents the issue of off-target radiation toxicity. The chief advantage of proton over proton radiation is the ability to conform to a narrower range of treatment depth without sacrificing treatment dose, thereby maximizing therapeutic effect and minimizing unintended side-scatter [34, 35]. The safety of hypofractionated pCSI in adults with solid tumor LM has been recently demonstrated in a phase 1b dose-expansion trial [36]. Of 20 evaluable patients treated with dose of 30 Gy in 10 fractions, the most common toxicities of any grade included fatigue (95%) and lymphopenia (90%), both of which were self-limiting in the majority of patients. No grade 3 gastrointestinal toxicities or cytopenias requiring blood transfusions were reported, suggesting more tolerable toxicity as compared to photon CSI. Importantly, treatment with pCSI was effective for both newly diagnosed and recurrent LM. Median overall survival (OS) was 8 months (95% *CI*: 6-NR) with a median CNS progression-free survival (PFS) of 7 months (95% *CI*: 5–13).

While small patient numbers and a mixed cancer population limits a more robust efficacy analysis of pCSI, the durability of disease control, seemingly agnostic to cancer type or mutational status, marks pCSI as deserving of further study. More investigation is needed to compare outcomes of pCSI to more conventional photon RT techniques for LM, determine optimal patient selection, and better characterize the short- and long-term toxicities associated with pCSI. A phase II study of pCSI versus involved-field photon RT in patients with solid tumor LM is ongoing (NCT04343573).

## Immunotherapy

Immune checkpoint inhibitors have demonstrated efficacy in the treatment of parenchymal brain metastases from melanoma [37, 38], lung adenocarcinoma [39–41], renal cell carcinoma [42–44], and to a lesser extent breast cancer [45, 46]. However, the activity of these agents within the leptomeningeal compartment appears less robust. While LM is marked by leukocytic pleocytosis, little is known about the migration of peripheral T-cells into intrathecal space [47]. T-lymphocytes within the leptomeninges in patients with LM are also immunologically distinct from matched brain metastases and generally harbor a more immunosuppressive phenotype [48]. Greater numbers of exhausted or dysfunctional CD4 and CD8 cells have been identified in the CSF in patients and animal models harboring LM, which therefore may not respond as consistently to immune checkpoint blockade [48, 49]. In fact, melanoma immunotherapy “non-responders” had consistently elevated numbers of inactivated T-lymphocytes despite changes in the myeloid cells, implying that while PD-1 therapy may alter the leptomeningeal microenvironment, this does not always translate to anti-tumor immunity [48].

Several studies have been conducted to define the role of intravenous immune checkpoint inhibition in patients with LM [50–54]. A phase II study tested intravenous pembrolizumab in 20 patients in LM from solid tumor malignancies [50]. While this study met its pre-specified primary endpoint with a median OS of 3.6 months (90% *CI*: 2.2–5.2 months), best CNS response was stable disease in 11 individuals with no patients demonstrating reduction in their LM burden. Combination ipilimumab and nivolumab for LM has also recently been studied in a phase II study of 18 patients, with a slightly shorter median OS of 2.9 months (90% *CI*: 1.6–5.0 months). However, this combination resulted in a complete response in 1 patient and stable disease in 8 patients [51]. Pooled CSF analysis from a subset of patients in these two trials revealed an increase in CD8+ T-lymphocytes and interferon-gamma signaling following immunotherapy administration [55]. This cytotoxic and antigen-processing signature tended to decrease with time, which may provide clues as to the relatively short duration of LM control. Further investigation is warranted to better understand the dynamic changes in the CSF following immune checkpoint inhibitor therapy, the relationship of these fluctuations with treatment response, and the factors that drive CSF T-lymphocytes into an activated rather than exhausted state. Additionally, both studies were enriched in patients with breast cancer. Analysis of these compounds in a higher number of patients with more immunotherapy-responsive malignancies, such as melanoma, might have revealed more durable outcomes. Combinatorial strategies to augment the bioactivity of immunotherapy in the leptomeningeal space are under investigation, including incorporation of WBRT (NCT03719768) [56] or targeted therapies (NCT04729348, NCT04511013, NCT04833205) into the treatment regimen.

There are multiple potential advantages of intrathecal as opposed to intravenous administration of these agents. First, if able to be administered into the intrathecal space safely, direct activation of CSF lymphocytes may provide a more potent and durable cytotoxic destruction of malignant cells in the spinal fluid. Second, directed immunotherapy may help to minimize the systemic autoimmune toxicities inherent to immune checkpoint blockade [57]. The concept of leptomeningeal-delivered immunotherapy has been previously studied in the form of intrathecal IL-2, a potent driver of effector T-cell differentiation [58]. Forty-three patients with melanoma LM treated with intrathecal IL-2 were found retrospectively to have a median survival of 7.8 months (range, 0.4–90.8 months), although treatment-related elevations in intracranial pressure were reported in all patients. Intrathecal administration of nivolumab is currently under investigation, both in combination with intravenous nivolumab in patients with melanoma LM (NCT03025256) and as monotherapy for patients with any solid tumor LM (NCT05112549). Intrathecal human epidermal growth factor receptor 2 (HER2)-chimeric antigen receptor (CAR) T cells, combining molecular targeting with immune-mediated therapy, is another potentially promising strategy still in the early phases of investigation (NCT03696030).

## Cancer-Specific Approaches

Systemic treatments of metastatic cancer have evolved considerably in the last two decades due to the incorporation of molecularly targeted treatments into first-line therapy, such as epidermal growth factor receptor (EGFR), anaplastic lymphoma kinase (ALK), and mesenchymal-epithelial transition (MET) pathway inhibitors for non-small-cell lung cancer (NSCLC) and HER2-active treatments for breast cancer. The extrapolation of such data to patients with CNS metastases has been largely limited to post hoc analyses in patients with stable or treated parenchymal brain metastases that were included in clinical trials. Less is known regarding the efficacy of such agents in patients with LM, though bioactivity and potential benefit has been suggested in several small reports.

### EGFR-Mutant NSCLC

Activating mutations in the *EGFR* gene, predominantly exon 19 deletions and exon 21 L858R mutations, are targetable oncogenic drivers of NSCLC [59, 60]. Patients harboring *EGFR*-mutant NSCLC are more likely to be never-smokers and are at increased risk of both brain and leptomeningeal metastases compared to *EGFR*-wildtype counterparts.

First- and second-generation EGFR tyrosine kinase inhibitors (TKIs), such as erlotinib, gefitinib, and afatinib, demonstrated activity in LM with detectable CSF concentrations at adequate dosing; however, durability of disease control was short-lived and partially explained by an acquired T790M resistance mutation [61–63]. Osimertinib, a third-generation TKI, selectively and irreversibly inhibits many *EGFR* mutations (including ex19del, L858R, T790M) and is now first-line therapy for patients with *EGFR*-mutant NSCLC irrespective of T790M status [64]. Osimertinib also demonstrates superior intracranial disease control and has shown promise in multiple clinical trials for patients with LM [65–68]. The AURA program allowed for inclusion of patients with asymptomatic or stable LM, and retrospectively calculated a LM-objective response rate (ORR) of 55% (95% *CI*: 32–76) to osimertinib 80 mg daily [65]. The BLOOM study, using a double dose of 160 mg daily to achieve higher CSF concentrations, prospectively determined a slightly higher LM-ORR of 62% (95% *CI*: 45–78) with a median OS of 11.0 months (95% *CI*: 8.0–18.0) [66]. A separate phase II analysis also indicated that LM progression while on osimertinib 80 mg may be halted with dose increase to 160 mg, though patient numbers were small and duration of response not reported [67]. To augment both intracranial and extracranial disease control, osimertinib is currently under investigation in combination with pemetrexed and platinum-based therapy (NCT04035486), which will include patients with brain metastases. Osimertinib in combination with bevacizumab, a vascular endothelial growth factor (VEGF) inhibitor, is also under investigation specifically in patients with LM (NCT04425681). Given the impressive disease control of TKIs in select NSCLC patients with targetable mutations, first-line TKI therapy has helped to postpone or even prevent CNS radiation in patients with LM. Despite durable responses of LM to third-generation EGFR inhibitors, acquired resistance inevitably occurs through both EGFR-dependent and EGFR-independent mechanisms [69]. Second-line therapies capable of overcoming osimertinib resistance are needed, with dedicated trials designed for patients with CNS metastases.

### MET Genomic Alterations in NSCLC

*MET* gene amplification is the most frequently encountered alternate bypass pathway conferring EGFR-TKI resistance, seen in 7–15% of patients with progression on first-line osimertinib [69]. To target this mechanism of resistance, combination of amivantamab, an EGFR-MET bispecific antibody with immune cell-directing activity, and lazertinib, a third-generation brain-penetrant EGFR inhibitor, is currently under investigation in both the newly diagnosed (MARIPOSA, NCT04487080) and recurrent (CHRYSALIS, NCT02609776) settings. While data from these trials are not yet mature [70], encouraging preliminary findings have led to the adaptation of combination amivantamab and lazertinib in patients with new or progressive CNS metastases harboring somatic activating *EGFR* mutations (NCT04965090). This phase II trial has been designed to enroll patients with parenchymal and leptomeningeal metastases in separate arms so as to best define the efficacy of this combination in different CNS compartments.

*MET* exon 14 skipping mutations occur in 3–4% of patients with NSCLC and are considered mutually exclusive with other oncogenic driver mutations, such as *EGFR*, *HER2*, and *KRAS* [71]. Capmatinib and tepotinib, highly selective inhibitors of the MET receptor, are both FDA-approved for patients with NSCLC harboring *MET* exon 14 skipping mutations and have demonstrated intracranial responses in clinical trials [72, 73]. Dramatic radiographic leptomeningeal responses have also been suggested in case reports of these two agents, with disease control for at least 5 months in one patient [74, 75].

### ALK-Rearranged NSCLC

*EML4-ALK* fusion mutations are identified in 3–13% of patients with NSCLC and are also enriched in the young, non-smoker population [76, 77]. The brain represents a common site of disease both at initial presentation and relapse; however, only approximately 5% of patients are known to develop LM as a delayed complication [78]. Both second-generation (ceritinib [79], alectinib [80], brigatinib [81]) and third-generation (lorlatinib [82–86]) ALK inhibitors have established superior intracranial efficacy compared to the first-generation ALK inhibitor (crizotinib [87]), though few protocols have specifically studied LM response rates of these individual agents. Improved intracranial control with later generation ALK inhibitors are a consequence of improved BBB and BCSFB penetration, with CSF concentrations approaching 75% that of plasma in the case of lorlatinib [88]. Lorlatinib, with dual ALK and ROS1 inhibition, gained FDA-approval for first line treatment of ALK-rearranged NSCLC following the interim results of the phase III CROWN study, confirming an intracranial ORR of 82% (95% *CI*: 57–96) among lorlatinib-treated TKI-naïve patients with measurable brain metastases [84].

Prospective data is sparse regarding the duration of LM control with lorlatinib treatment. A prospective phase II study of lorlatinib in patients previously treated with TKIs enrolled 2 patients with ALK+ LM at baseline: one achieved a complete intracranial response for 21.9 months and the second achieved a partial intracranial response for 11 months [83]. The largest dataset of lorlatinib activity in patients with LM comes from an international early/expanded access program of 95 patients, including 13 patients with LM (11 ALK+ and 2 ROS1+) who had progressed on prior TKI therapy [89]. Intracranial ORR in the LM cohort was 45% (95% *CI*: 17–77) with a disease control rate of 91% (95% *CI*: 59–100) among 11 evaluable patients. Two patients in the ALK+ cohort had a complete response intracranially. Median PFS was 9.3 months (95% *CI*: 1.0–NR) in the entire LM cohort. Investigation of a German early access program revealed a partial response rate of 77.8% among 9 patients with LM, but did not provide further details regarding duration of response [90]. While these expanded access datasets are limited due to their retrospective nature and lack of protocolized neuraxial assessments, they complement a number of case reports demonstrating improvement of both LM disease burden and neurologic symptoms following lorlatinib treatment [91–93].

### HER2-Positive Breast Cancer

Targeted therapies for patients with LM from breast cancer have been mainly restricted to HER2-active agents with adequate CNS penetration. Trastuzumab, while the backbone of combination therapy in patients with HER2+ breast cancer, has negligible CNS penetration which results in high rates of intracranial relapse in this patient population [94]. Approximately 25–50% of HER2+ patients treated with trastuzumab will develop brain metastases and 6–7% will develop LM [95].

While an intrathecal formulation of trastuzumab seems to be well-tolerated across a number of prospective and retrospective studies, conclusions regarding its efficacy have been mixed. A multicenter phase I/II trial determined intrathecal trastuzumab to have minimal toxicity with disease control in 69% of patients with HER2+ breast cancer and LM [96, 97]. However, the primary endpoint (25% RR) was not met and the median PFS was 2.4 months (95% *CI* 1.0–5.5). A single institution retrospective review of breast cancer LM treatments found a 6-month CNS-PFS of 44% for intrathecal trastuzumab, 18% for intrathecal chemotherapy, and 26% for WBRT. OS was also longer among the intrathecal trastuzumab cohort (54% alive at 12 months compared to 10% for intrathecal chemotherapy and 19% for WBRT, respectively), although confounded by the enrichment of systemic disease and triple-negative breast cancer among the intrathecal chemotherapy and WBRT cohorts [98]. A subsequent meta-analysis of intrathecal trastuzumab across 24 articles calculated a CNS-PFS of 5.2 months (*N* = 39) and median OS of 13.2 months (*N* = 54) [99]. Further randomized controlled studies are needed to fully validate the clinical efficacy of intrathecal trastuzumab. The use of WBRT and/or focal RT followed by intrathecal trastuzumab and pertuzumab is currently being investigated in HER2+ LM patients (NCT04588545).

Small molecule HER2-TKIs, namely lapatinib, neratinib, and tucatinib, offer an orally available adjuvant treatment to HER2+ breast cancer patients at the development of brain metastases. Lapatinib and neratinib each confer better intracranial control when in combination with capecitabine compared to monotherapy [100–104]. A few pharmacokinetic series have reported very low concentrations of neratinib and lapatinib in the CSF relative to plasma [105, 106]. Nevertheless, small prospective studies and case reports suggest possible activity of these combinations in patients with LM [103, 104, 107]. A single phase II study investigating neratinib and capecitabine, including 3 patients with LM, found stable and partial responses in 2 patients with disease control of 4 and 7 months, respectively [103]. A phase I study series of intermittent high-dose lapatinib plus capecitabine included 5 patients with LM [104]. Of the 4 patients who completed at least 1 cycle of treatment, 2 patients achieved partial and stable responses and remained on study for at least 6 months.

Tucatinib, a highly-selective third-generation HER2-TKI, demonstrated compelling intracranial activity in the HER2CLIMB trial, which evaluated tucatinib or placebo in combination with trastuzumab and capecitabine in patients with previously-treated HER2+ breast cancer [108]. Exploratory analysis of patients enrolled with active or stable brain metastases revealed a significant improvement in CNS-PFS (9.9 months versus 4.2 month) and median OS (18.1 versus 12.0 months) with the addition of tucatinib [109]. While patients with LM were excluded from this study, these encouraging results prompted the development of a phase II trial of combination tucatinib, trastuzumab, and capecitabine in patients with HER2+ breast cancer and LM (NCT03501979) [110]. Preliminary mass spectrometric analysis of paired CSF and blood samples from this patient cohort suggests detection of both tucatinib and its metabolite, ONT-993, in the CSF [111].

Two FDA-approved antibody–drug conjugates, trastuzumab emtansine (T-DM1) and trastuzumab deruxtecan (T-DXd), facilitate receptor-mediated endocytosis of cytotoxic payloads into HER2+ cells. Exploratory analysis of patients with baseline brain metastases in the phase IIIb KAMILLA and phase III EMILIA studies of T-DM1 found a median PFS of 5.5–5.9 months and median OS of approximately 2 years [112, 113]; however, no results regarding leptomeningeal activity were discussed and only 1 case report to date comments on the activity of T-DM1 in combination with WBRT in a patient with intracranial LM [114]. T-DXd, conversely, offers more potent cytotoxic activity with a higher drug-antibody ratio, use of a potent topoisomerase I compared to a microtubule inhibitor, and a membrane-permeable payload capable of “bystander” activity against HER2-negative or HER2-low cells [115]. While data regarding T-DXd efficacy in LM is not available, an encouraging median PFS of 18.1 months (95% *CI*: 6.7–18.1) was calculated among patients with brain metastases in the phase II DESTINY-Breast01 study [116]. Several clinical trials of T-DXd for HER2+ cancers are now underway, including the phase II TUXEDO-1 (NCT04752059) [117], DEBBRAH (NCT04420598), and DESTINY-B12 (NCT04739761) studies designed for patients with brain metastases. Importantly, the DEBBRAH study includes a cohort specifically for patients with LM.

### Hormone Receptor-Positive Breast Cancer

While endocrine therapy is routinely employed in breast cancer patients with estrogen and progesterone receptor positivity, data regarding hormonal agents in LM are lacking. No prospective clinical trial addresses this question directly for patients with hormone receptor (HR)-positive breast cancer. There is evidence that tamoxifen can concentrate within parenchymal brain metastases; it is also detected at very low levels in the CSF [118, 119]. Preclinical models suggest that letrozole, and to a lesser extent, anastrozole, penetrate the BBB, but it is unknown whether either agent accesses the CSF [120]. This distinction may not be clinically relevant in the case of aromatase inhibitors, as these agents act on peripheral estrogen production as opposed to direct blockade of estrogen receptors. Despite the uncertainty regarding tumor-directed efficacy of hormonal agents in LM, it is worth noting that the use of endocrine therapy after brain or leptomeningeal metastasis diagnosis has been associated with prolonged survival, with no difference identified between choice of endocrine therapy employed [121]. This finding is supported also by a number of case reports suggesting durable response of LM to several hormonal agents, with most cases describing success with letrozole, exemestane, and tamoxifen [122–126].

Resistance to endocrine therapies can occur through both cell-cycle dysregulation and upregulation of the phosphatidylinositol 3-kinase (PI3K)/protein kinase B (AKT)/mammalian target of rapamycin (mTOR) pathway [127]. Combining hormonal agents with an inhibitor of these pathways represents an attractive option mechanistically with meaningful systemic disease control [128–131]. The bioactivity of such regimens against HR+ brain and leptomeningeal metastases has been suggested in small prospective studies and case reports, although objective responses tend to be small. In phase II study of abemaciclib, a CNS-penetrant cyclin-dependent kinase (CDK) 4/6 inhibitor, with or without endocrine therapy in patients with HR+ breast cancer brain and leptomeningeal metastases, abemaciclib concentrations were roughly equivalent in the plasma and CSF [132]. The intracranial benefit rate was 24% among HR+ HER2− patients. In the LM cohort, 3 of 7 patients with HR+ LM achieved stable disease as best response, with 1 patient experiencing disease control for > 6 months. Combined PFS was 5.9 months (95% *CI*: 0.7–8.6) and median OS was 8.4 months (95% *CI*: 3.3–23.5) for the HR+ HER2− LM patients.

No clinical trials to date have reported on the use of PI3K/AKT/mTOR inhibition in patients with breast cancer LM. Alpelisib, an FDA-approved selective PI3Kα inhibitor, has shown modest brain metastasis control in case reports, despite a paucity of data regarding BBB and BCSFB penetration [133]. The brain- and CSF-penetrant pan-PI3K inhibitor, buparlisib, has also demonstrated brain metastasis activity, but its use has been limited by toxicities including transaminitis and hypergylcemia [134–137]. Paxalisib, a dual PI3K and mTOR inhibitor, is currently under investigation in combination with radiation in patients with LM (NCT04192981).

### Triple Negative Breast Cancer

The treatment of CNS metastases from triple negative breast cancer (TNBC) is fraught with challenges, both due to the paucity of targetable agents for these patients and the high frequency with which metastatic disease to the CNS occurs in this population [138]. Consequently, patients with TNBC with brain and leptomeningeal metastases suffer inferior outcomes [139].

Breast cancer patients harboring germline *BRCA1* mutations are more likely to express the triple-negative phenotype than those with *BRCA2* mutations or *BRCA*-noncarriers [140, 141]. *BRCA1*-mutant patients also tend toward higher nuclear grade and tumor burden [140, 141]. Olaparib and talazoparib, two FDA-approved poly ADP ribose polymerase (PARP) inhibitors for breast cancer patients with germline *BRCA* mutations [142, 143], demonstrate clinical activity against brain metastases [144, 145]. While there are limited data regarding CSF penetration of these agents, durable near-complete responses of LM to olaparib monotherapy have been reported, with disease control lasting >12 months [146–148]. Additionally, olaparib resulted in improvement in symptoms and quality of life in these heavily pre-treated patients.

Repurposing of existing chemotherapies to augment brain and CSF penetration is another potential mechanism to improve disease control in TNBC LM patients. For example, paclitaxel has very poor BBB penetration. However, when conjugated with Angiopep-2, an amino acid sequence that binds low-density lipoprotein receptor-related protein-1 at the BBB and BCSFB, paclitaxel demonstrates improved intrathecal penetration [149]. This agent, termed ANG1005 (paclitaxel trevatide), was studied in a phase II trial in patients with breast cancer brain and leptomeningeal metastases, demonstrating an intracranial ORR of 17% (95% *CI*: 4.7–37.4), intracranial clinical benefit rate of 79% (95% *CI*: 57.8–92.9), and median PFS of 14.9 weeks (95% *CI*: 12.7–23.4) among those with LM [149]. Survival rate varied by breast cancer subtype, with a median OS of 9.0 months (95% *CI*: 5.4–15.2) for HER2+ LM, 7.6 months (95% *CI*: 1.4–9.4) for HER2− LM, and 2.8 months (95% *CI*: 0.8–8.7) for TNBC LM. A phase III study of ANG1005 in HER2− breast cancer patients has been designed (NCT03613181).

Finally, capecitabine is an oral prodrug metabolized into the antimetabolite, 5-fluorouracil, and therefore is agnostic to HR and HER2 status in its mechanism of action. Case reports suggest activity of capecitabine against both HR+ and TNBC LM [150–153]. Intravenous high-dose methotrexate can also be considered for patients with adequate organ function and can obviate the need for intrathecal methotrexate [154].

### Melanoma

Melanoma is notorious for CNS dissemination, estimated to occur in 50–75% of patients as per autopsy series [155, 156]. In fact, patients with de novo metastatic melanoma have the highest incidence of brain metastases at diagnosis than any other malignancy [157]. The frequency of LM in melanoma patients ranges from 5 to 30% [158, 159]. Immunotherapy has revolutionized the treatment of melanoma brain metastases, resulting in a 3-year survival of 72% and 37% for those with baseline asymptomatic and symptomatic metastases, respectively [37, 38]. Approximately 40% of melanoma patients harbor a *BRAF* V600 driver mutation, making them attractive targets of BRAF and MEK inhibitors upon immunotherapy failure. Combination dabrafenib/trametinib and encorafenib/binimetinib both achieve intracranial disease control in patients with melanoma brain metastases, although the duration of intracranial responses tends to be short-lived [160, 161].

The combination of immune checkpoint inhibitors with LM-directed radiation have resulted in durable melanoma LM control in a few case reports [162–165], but these results have not been recapitulated in prospective studies. One phase II study of ipilimumab and nivolumab in patients with melanoma CNS metastases included 4 patients with LM, none of whom achieved a response [166]. Data regarding intrathecal administration of nivolumab in patients with melanoma is not yet mature (NCT03025256) [167], but if proven successful, might circumvent many of the systemic toxicities associated with immune checkpoint blockade. Other intrathecal immunotherapeutic approaches have achieved durable responses of melanoma LM, including tumor-infiltrating lymphocytes [168] and cytotoxic T-lymphocytes [169]. Prospective clinical investigations are needed to better evaluate these methods in a larger cohort of patients.

There have been no prospective trials to date regarding the efficacy of BRAF and MEK inhibition for melanoma LM. A number of case reports suggest that these agents may be effective against LM, either alone, in combination with immunotherapy, or following LM-directed radiation [165, 170–175]. However, despite clear intracranial activity of a number of BRAF and MEK inhibitors, these agents are often substrates to the P-glycoprotein and breast cancer resistance protein drug efflux pumps, with suboptimal intracranial penetration the likely result [176]. The CSF penetration of these agents has not been well characterized. For example, the BRAF inhibitor, vemurafenib, has a low CSF penetration rate with high interindividual variability and no clear correlation with plasma levels [177]. Further exploration of the CSF activity of these agents, and development of reliably CNS-penetrant BRAF/MEK inhibitors, is needed. A phase I first-in-human study of a novel CNS-penetrant BRAF inhibitor, PF-07284890, in combination with binimetinib is underway in patients with intracranial *BRAF* V600 mutant solid tumor (NCT04543188). Encouragingly, this basket trial includes a cohort for patients with LM. A phase II study of encorafenib, binimetinib, and nivolumab versus ipilimumab and nivolumab in *BRAF* V600 mutant melanoma patients with brain and leptomeningeal metastases is also currently recruiting (NCT04511013).

## Novel Treatments

One of the principal barriers in treating LM is the paucity of knowledge on cancer cell survival in the nutrient-sparse CSF [178]. Unlike metastases to other sites of the body, LM thrive in an anatomic compartment devoid of a blood-tumor capillary network and therefore must acquire nutrients by other means. Identification and depletion of these critical micronutrients in the CSF would place further constraints on LM metabolism and might provide a unique, targeted mechanism of LM treatment. Free iron within the CSF is one such nutrient, found in higher concentrations in the CSF of LM patients compared to non-LM controls [179]. Cancer cells in the CSF uniquely employ a single iron-binding transporter and receptor, lipocalin-2 and SLC22A17, to scavenge iron and sustain their metabolic needs. Exploitation of cancer cell’s dependency on iron using intrathecal administration of an iron chelator, deferoxamine, in preclinical LM models resulted in reduction in CSF iron and diminished LM growth. Furthermore, this reduction in LM burden correlated with significant prolongation of survival in mice. IT-DFO was well tolerated in these preclinical studies, without undue neurologic toxicity. A phase 1a/b dose escalation and expansion study of intrathecal deferoxamine is currently underway in patients with LM from solid tumor malignancies (NCT05184816).

Targeted radioimmunotherapy represents another unique treatment modality for LM, thus far tested most heavily in pediatric patients [180]. 177Lu-omburtamab-DTPA is an intrathecally-administered monoclonal B7H3 antibody labeled with lutetium-177 to deliver focused radioimmunotherapy to tumor cells. B7H3 belongs to the B7 family of immune checkpoint molecules, and its aberrant overexpression in many tumor tissues is a newly recognized marker for metastatic progression [181]. This investigational compound was granted rare pediatric disease designation in 2021 by the FDA, and is currently being evaluated in children with recurrent or refractory medulloblastoma (NCT04167618).

Brachytherapy using liposomally encapsulated radionucleotides is another strategy under investigation for both solid tumor LM and recurrent glioblastoma. Rhenium-186 NanoLiposomes (186RNL), either through intraventricular administration or convection-enhanced delivery, allow for the delivery of beta-emitting radiation with high specificity for CNS tumors and with relative sparing of normal brain tissue [182, 183]. The ReSPECT-LM trial, a phase I dose-finding study of intraventricularly-administered 186RNL in patients with LM, is currently open and enrolling (NCT05034497).

## Considerations in LM Clinical Trial Design

The scientific community and organizations responsible for improving outcomes in patients with cancer have made tremendous progress in advocating for CNS-directed clinical trials. Continued development of protocols designed specifically for patients with LM is essential to moving the needle for this underserved population, particularly as the incidence of LM is expected to increase. Important nuances regarding clinical trial design for patients with LM require thoughtful consideration.

First, clinical protocols investigating LM lack standardization and response assessments tend to be unvalidated and time-consuming. Discordant changes in cytologic and radiographic responses can occur, complicated even further by the poor sensitivity of CSF cytology and inability to sufficiently quantify smooth linear leptomeningeal deposits. Most protocols employ modifications of the Response Assessment in Neuro-Oncology (RANO) and European Association of Neuro-Oncology—European Society for Medical Oncology (EANO-ESMO) consensus guidelines [7, 8]. Continued collaborative efforts between consensus groups such as these are essential to provide uniformity and validity to LM response assessments.

Proper clinical endpoint selection in LM clinical trial design is critical in making accurate conclusions regarding therapeutic benefit. Determination of objective responses in LM is challenging due to reasons listed above and may be unreliable in the context of multimodal response assessments. As a result, many clinical protocols choose the primary endpoint of OS in effort to maintain stringency in clinical trial reporting, with comparison to historical control survival data as a marker of success. However, as modern therapies in the last decade improve patient survival, particularly among those with available molecular targets, the historical survival benchmark of 3–4 months may not be the best comparator for all LM subtypes. Furthermore, inclusion of patient-reported outcomes and quality of life assessments are essential to ensure that emerging treatments are not only efficacious but also ameliorate neurologic morbidity.

Finally, CSF circulating tumor cell (CTC) enumeration and circulating tumor DNA analyses represent important quantitative and qualitative biomarkers of LM [97, 184–186]. These techniques aim to improve diagnostic sensitivity, identify molecular drivers of site-specific progression and therapeutic resistance, and provide important prognostic and predictive insights [97]. Incorporation of these techniques into clinical trial design may help to optimize patient selection and finesse LM response assessments.

While optimal enrollment strategies, endpoint determination, and biomarker selection in patients with LM require continued refinement, the expansion of LM-directed clinical trials in the last decade is highly encouraging. Clinicians must consider that most conventional treatments for this patient population, such as WBRT and intrathecal therapy, have failed to demonstrate a survival benefit in controlled prospective studies. Therefore, enrollment into therapeutic protocols is paramount to adequately investigate newer translational approaches, and clinical trials should be prioritized whenever possible.

## Conclusion

The recent emergence of clinical trials designed specifically for both brain and leptomeningeal metastasis patients is an encouraging testament to scientific progress and patient advocacy. Proton CSI is safe and effective in the treatment of LM, and several modern therapies demonstrate bioactivity in the CSF in small prospective studies and case reports. Discordant drug responses between the brain and leptomeningeal compartments should trigger further investigation, including dedicated clinical protocols designed for LM, pharmacokinetic monitoring to ensure adequate CSF penetration, and biomarker analysis for potential pathways of therapeutic resistance. Further characterizations of the leptomeningeal immune microenvironment and the molecular mechanisms that underlie LM progression have the potential to uncover novel treatments for this underserved patient population.

## Supplementary Information

Below is the link to the electronic supplementary material.

Supplementary file1 (DOCX 35 kb)

Supplementary file2 (DOCX 33 kb)

Supplementary file3 (DOCX 33 kb)
