# Supplementary material for: Leptomeningeal Metastases: New Opportunities in the Modern Era
Source: Neurotherapeutics. 2022 Jul 5;19(6):1782–98. doi: 10.1007/s13311-022-01261-4 (PMC9723010; doi:10.1007/s13311-022-01261-4)
Supplement: Supplementary file 1 — Supplementary file1 (DOCX 35 kb) [file 13311_2022_1261_MOESM1_ESM.docx]

| ICMJE DISCLOSURE FORM | |
| --- | --- |
| **Date:** | 2/28/2022 |
| **Your Name:** | Adrienne A. Boire |
| **Manuscript Title:** | Leptomeningeal Metastasis: New opportunities in the Modern Era |
| **Manuscript Number (if known):** | Click or tap here to enter text. |
| In the interest of transparency, we ask you to disclose all relationships/activities/interests listed below that are related to the content of your manuscript. “Related” means any relation with for-profit or not-for-profit third parties whose interests may be affected by the content of the manuscript. Disclosure represents a commitment to transparency and does not necessarily indicate a bias. If you are in doubt about whether to list a relationship/activity/interest, it is preferable that you do so.  The author’s relationships/activities/interests should be defined broadly. For example, if your manuscript pertains to the epidemiology of hypertension, you should declare all relationships with manufacturers of antihypertensive medication, even if that medication is not mentioned in the manuscript.  In item #1 below, report all support for the work reported in this manuscript without time limit. For all other items, the time frame for disclosure is the past 36 months. | |

|  | | | **Name all entities with whom you have this relationship or indicate none (add rows as needed)** | **Specifications/Comments (e.g., if payments were made to you or to your institution)** |
| --- | --- | --- | --- | --- |
| **Time frame: Since the initial planning of the work** | | | | |
| **1** | All support for the present manuscript (e.g., funding, provision of study materials, medical writing, article processing charges, etc.)  **No time limit for this item.** | | \|  \| **None** \| \| --- \| --- \|  \| NIH/NCI \| Cancer Center Support Grant P30 CA008748 \| \| --- \| --- \| | |
| **Time frame: past 36 months** | | | | |
| **2** | | Grants or contracts from any entity (if not indicated in item #1 above). | \|  \| **None** \| \| --- \| --- \|  \| The Pew Charitable Trusts \| “Dissecting Cancer Cell Evolution in Leptomeningeal Metastasis” 2018-2022 \| \| --- \| --- \| \| Pershing Square Sohn Cancer Research Alliance \| “Molecular Dissection of Innate Immunity in Leptomeningeal Metastasis” 2019-2022 \| \| Joe W. and Dorothy Dorsett Brown Foundation \| “Defining and Dissecting Immune Cancer Cell Interactions in the Spinal Fluid”2019-2021 \| \| W.M. Keck Foundation \| “The Choroid Plexus as Premetastatic Niche for Leptomeningeal Metastasis” 2019-2022 \| \| American Brain Tumor Association \| “Immunological Determinants of Metastatic Colonization of Leptomeninges” 2019-2021 \| \| Alan and Sandra Gerry Metastasis and Tumor Ecosystems Center \| “Microenvironmental Effects on Cancer Cells in Leptomeningeal Metastasis” 2019-2021 \| \| AACR \| “The Microenvironmental Landscape of Breast Cancer Leptomeningeal Metastasis” \| \| National Institutes of Health \| “Investigating Microenvironmental Interactions in Leptomeningeal Metastasis” 1 R01 CA245499-01A1 **2020-2025** \| \| STARR Cancer Consortium \| “Capture of Leptomeningeal Cancer Cell and Macrophage Iron Metabolism” **2021-2024** \| \| MSKCC Center for Experimental Therapeutics \| “Phase 1a/1b Trial to Assess Safety and Bioactivity of Intrathecal Deferoxamine in Patients with Leptomeningeal Metastases from Non-Small Cell Lung Cancer” 2021-2023 \| \| FM Kirby Foundation \| “Phase 1a/1b Trial to Assess Safety and Bioactivity of Intrathecal Deferoxamine in Patients with Leptomeningeal Metastases from Non-Small Cell Lung Cancer” 2021-2023 \| \|  \|  \| | |
| **3** | | Royalties or licenses | \|  \| **None** \| \| --- \| --- \|  \|  \|  \| \| --- \| --- \| \|  \|  \| \|  \|  \| | |
| **4** | | Consulting fees | \|  \| **None** \| \| --- \| --- \|  \|  \|  \| \| --- \| --- \| \|  \|  \| \|  \|  \| \|  \|  \| | |
| **5** | | Payment or honoraria for lectures, presentations, speakers bureaus, manuscript writing or educational events | \|  \| **None** \| \| --- \| --- \|  \|  \|  \| \| --- \| --- \| \|  \|  \| \|  \|  \| | |
| **6** | | Payment for expert testimony | \|  \| **None** \| \| --- \| --- \|  \|  \|  \| \| --- \| --- \| \|  \|  \| \|  \|  \| | |
| **7** | | Support for attending meetings and/or travel | \|  \| **None** \| \| --- \| --- \|  \| Forbeck Forum on Cellular Reprogramming and Metastatic Disease \| “Cancer Cells Employ Astrocytes as Energetic Source in Leptomeningeal Metastasis”. 2021 December 3; Denver, CO, United States. \| \| --- \| --- \| \| Melanoma Research Foundation \| “Inflammatory Cytokines Support Cancer Cell Growth in the Leptomeninges”. 2021 November 11; Washington DC, United States. \| \| Stanford University Department of Neurology \| “Inflammatory Pathologies of the Leptomeninges: from Cancer to COVID-19” Grand Rounds. 2021 November 5; Palo Alto, CA, United States. \| \| Gordon Research Conference on the Cell Biology of Metals \| “Cancer Cells Outcompete Macrophages for Sparse Extracellular Iron in the Leptomeninges”. 2021 October 19; Mt. Snow, VT, United States \| \| Washington University Siteman Cancer Center: Cell-to-Cell Communications in Cancer Program \| “Overcoming Metabolic Constraints in Leptomeningeal Metastasis”. 2019 December 11; Saint Louis, MO, United States. \| | |
| **8** | | Patents planned, issued or pending | \|  \| **None** \| \| --- \| --- \|  \| Sloan Kettering Institute, assignee \| Boire A and J Massagué, inventors. Sloan Kettering Institute, assignee. Modulating Permeability Of The Blood Cerebrospinal Fluid Barrier. United States Provisional Application No.: 62/258,044. November 20, 2015. \| \| --- \| --- \| \| Sloan Kettering Institute, assignee \| Boire A, Chen Q and J Massagué, inventors. Sloan Kettering Institute, assignee. Methods for Treating Brain Metastasis. United States 10413522, awarded September 17, 2019. \| \| Sloan Kettering Institute, assignee \| Boire A, inventor. Sloan Kettering Institute, assignee. Methods of Treating Leptomeningeal Metastasis. United States Provisional Application No.: 63/052,139. Jul 15, 2020 \| | |
| **9** | | Participation on a Data Safety Monitoring Board or Advisory Board | \|  \| **None** \| \| --- \| --- \|  \|  \|  \| \| --- \| --- \| \|  \|  \| \|  \|  \| | |
| **10** | | Leadership or fiduciary role in other board, society, committee or advocacy group, paid or unpaid | \|  \| **None** \| \| --- \| --- \|  \| Evren Scientific \| Scientific Advisory Board (unpaid) \| \| --- \| --- \| \|  \|  \| \|  \|  \| | |
| **11** | | Stock or stock options | \|  \| **None** \| \| --- \| --- \|  \|  \|  \| \| --- \| --- \| \|  \|  \| \|  \|  \| | |
| **12** | | Receipt of equipment, materials, drugs, medical writing, gifts or other services | \|  \| **None** \| \| --- \| --- \|  \|  \|  \| \| --- \| --- \| \|  \|  \| \|  \|  \| | |
| **13** | | Other financial or non-financial interests | \|  \| **None** \| \| --- \| --- \|  \|  \|  \| \| --- \| --- \| \|  \|  \| \|  \|  \| | |
|  | |  |  | |
| **Please place an “X” next to the following statement to indicate your agreement:** | | | | |
|  | | I certify that I have answered every question and have not altered the wording of any of the questions on this form. | | |
